# Immunogenicity of recombinant *Mycobacterium bovis* bacille Calmette–Guèrin clones expressing T and B cell epitopes of *Mycobacterium tuberculosis* antigens

**DOI:** 10.1186/1471-2172-14-S1-S5

**Published:** 2013-02-25

**Authors:** Rohimah Mohamud, Maryam Azlan, Daniel Yero, Nadine Alvarez, Maria E Sarmiento, Armando Acosta, Mohd-Nor Norazmi

**Affiliations:** 1 School of Health Sciences, Universiti Sains Malaysia, 16150 Kubang Kerian, Kelantan, Malaysia; 2 Finlay Institute, Molecular Biology Department, Ave 27 No. 19805. La Lisa. PC 11600, La Habana, Cuba; 3 Institute for Research in Molecular Medicine, Universiti Sains Malaysia, 16150 Kubang Kerian, Kelantan, Malaysia

## Abstract

Recombinant *Mycobacterium bovis* bacille Calmette–Guèrin (rBCG) expressing three T cell epitopes of *Mycobacterium tuberculosis* (MTB) Ag85B antigen (P1, P2, P3) fused to the Mtb8.4 protein (rBCG018) or a combination of these antigens fused to B cell epitopes from ESAT-6, CFP-10 and MTP40 proteins (rBCG032) were used to immunize Balb/c mice. Total IgG responses were determined against Mtb8.4 antigen and ESAT-6 and CFP-10 B cell epitopes after immunization with rBCG032. Mice immunized with rBCG032 showed a significant increase in IgG1 and IgG2a antibodies against ESAT-6 and MTP40 (P1) B cell epitopes and IgG3 against both P1 and P2 B cell epitopes of MPT40. Splenocytes from mice immunized with rBCG018 proliferated against Ag85B P2 and P3 T cell epitopes and Mtb8.4 protein whereas those from mice-immunized with rBCG032 responded against all Ag85B epitopes and the ESAT-6 B cell epitope. CD4^+^ and CD8^+^ lymphocytes from mice immunized with rBCG018 produced primarily Th1 type cytokines in response to the T cell epitopes. Similar pattern of recognition against the T cell epitopes were obtained with rBCG032 with the additional recognition of ESAT-6, CFP-10 and one of the MTP40 B cell epitopes with the same pattern of cytokines. This study demonstrates that rBCG constructs expressing either T or T and B cell epitopes of MTB induced appropriate immunogenicity against MTB.

## Introduction

Tuberculosis (TB) is one of the most prevalent diseases in developing countries. WHO estimates that 8.7 million new cases and approximately 1.6 million deaths occur annually [1,2] . The current vaccine against *Mycobacterium tuberculosis*, *M. bovis* bacille Calmette–Guèrin (BCG), is the most widely used vaccine in preventing TB disease especially in childhood [3,4] . However, it has also been established that the protection afforded by BCG is highly controversial [5] . Thus, a more effective TB vaccine is urgently needed.

It has been reported that there was only a slight decrease in the protective efficacy of BCG after 50 years [6] . The intrinsic advantage of BCG as a live vector is its capability to replicate in host macrophages. In addition, BCG can stimulate both cellular and antibody responses and has the possibility to stimulate prolonged memory T cell responses [7] . Moreover, BCG is safe, stable and inexpensive and thus is an attractive target for genetic manipulation in order to improve its protective capability against TB [8] . BCG also represents an effective vehicle for delivery of heterologous antigens due to its intracellular replication in macrophages and dendritic cells [9].

Despite the dominant dogma that the cellular immune mechanism is primarily responsible for protection against TB, recent evidences suggest a protective role of specific antibodies in the defense against intracellular microorganisms such as MTB [10-14] . There is also an agreement that no single mycobacterial protein or antigen will be able to evoke all the required immune response for effective protection [15] . Vaccine formulations encoding multivalent mixtures of antigens are necessary to increase the chances for covering populations with different MHC [16] .This also may stimulate the innate immune system in providing adequate cytokine and costimulatory molecules [17] . Thus, the multiantigen or multiepitope approach is an important strategy that needs to be explored.

In this study, T and B cell epitopes of MTB were cloned into BCG. The immunogenicity of the rBCG expressing three T cell epitopes of the Ag85B antigen of MTB fused to the entire sequence of MTB8.4 protein and the same construct fused to B cell epitopes of ESAT-6, CFP-10 and MTP-40 proteins of MTB were evaluated to determine its ability to induce specific humoral and cellular immune responses against these epitopes.

## Materials and methods

### Design of the multiepitope MTB gene fragments

The sequences of the MTB proteins were obtained from the UNIPROT database [18]. Linear MTB B cell epitopes were predicted from CFP-10 [18], ESAT-6 [19], and MTP40 [20] proteins using the BCEPRED software [21]. T cell epitopes were selected from MTB Ag85B protein based on a previous report [22] and analyzed using the prediction program PredBalb/c [23].

### Construction of rBCG clones

Two multiepitopic constructs were designed: one containing three T cell epitopes of Ag85B protein of MTB fused to the Mtb8.4 protein, cloned into the shuttle plasmid pNMN018 and another with the addition of the selected B cell epitopes upstream to the previous construct, contained in the shuttle plasmid pNMN032. Both constructs were generated using preferred mycobacterium codon usage.

### Immunization of Balb/c mice with rBCG

Balb/c mice (*n* = 5 per group) were immunized intraperitoneally (i.p.) with 2 x 10^6^ CFU of either rBCG018 or rBCG032 in 200 μl PBS containing 0.1% Tween 80 (PBS-T80). A control group of mice were injected with 2 x 10^6^ CFU of the parental BCG strain in 200 μl PBS-T80. After 30 and 60 days, the same amount of either rBCG or parental BCG was injected i.p. as boosters. Sera were collected before each injection from the tail vein and kept at -20 °C for IgG antibodies measurement. Subsequently, three weeks after the last immunization the mice were sacrificed and the spleens were asseptically removed to assess the cellular immune response.

### Construction of peptides

The peptides corresponding to the T and B cell epitopes were commercially prepared (Peptron, Korea) and the Mtb8.4 antigen was produced in *E. coli*.

### Enzyme-linked immunosorbent assay (ELISA)

Total IgG and IgG1, IgG2a, IgG2b and IgG3 (only against B cell epitopes) were measured from sera of immunized mice by ELISA as described elsewhere. The results were analysed using One-way ANOVA test and the differences between pairs were determined by the Multiple Range test. *p* values less than 0.05 were considered statistically significant.

### Lymphocyte proliferation assay

T and B cell epitopes or recombinant Mtb8.4 protein were added to the cultures at a final concentration of 10 μg/ml. RPMI medium was used as negative control and the T cell mitogen, concanavalin A (10 μg/ml) was used as positive control. Results were calculated as stimulation index (SI) (SI = cpm of stimulated culture/cpm of non-stimulated culture). SI of more than 2 was considered as positive.

### Analysis of intracellular cytokines

10 μg/ml of each peptide corresponding to the T and B cell epitopes or recombinant Mtb8.4 protein were cultured with 2 × 10^6^ spleen cells/ml. The cells were then stained with 20 μl (20 μg /ml) anti-mouse IL-2, IL-4 or IFN-γ labelled with PE (BD Pharmingen). The increase in the percentage of intracellular cytokine levels in lymphocytes of rBCG-immunized mice was compared to those of BCG-immunized mice and scored as high, moderate, or weak/absent corresponding to ≥ 3 fold, ≥ 2 fold, < 2 fold increase respectively.

## Results and discussion

### Antibody response against T and B cell epitopes

**Figure 1:**
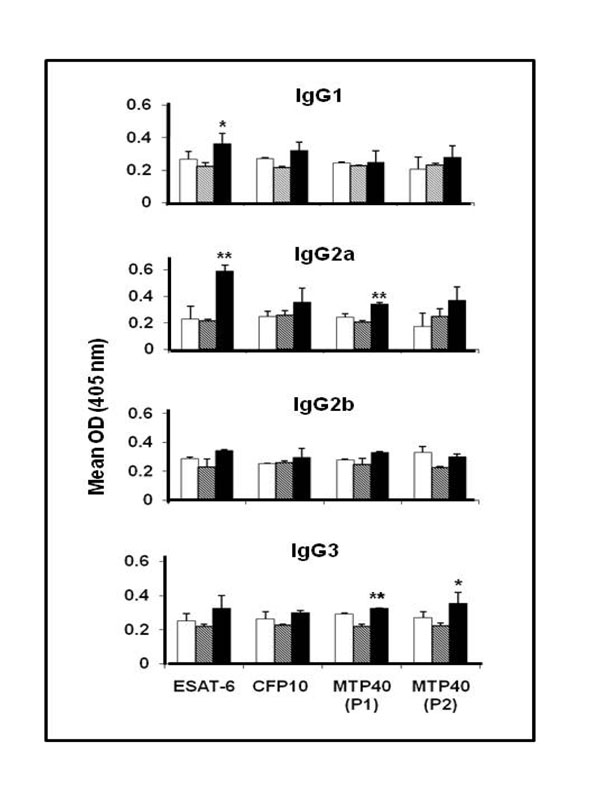
Profile of antigen-specific IgG subclasses response against ESAT-6, CFP-10, MTP40 (P1) and MTP40 (P2) B cell epitopes in Balb/c mice (n = 5 for each group) immunized with BCG (open bars) rBCG018 (hatched bars), and rBCG032 (solid bars). The results are expressed as mean OD at 405 nm. * p < 0.05; ** p < 0.01

Significant increase in the level of total IgG against ESAT-6 and CFP-10 B cell epitopes was observed after the third immunization of mice with rBCG032 compared to other groups. Increased levels of specific IgG1 were detected against ESAT-6 (p < 0.05); IgG2a against ESAT-6 (p < 0.01) and MTP40 (P1) (p < 0.01); and IgG3 against MTP40 (P1) (p < 0.01) and MTP40 (P2) (p < 0.05) in mice immunized with rBCG032 compared to other groups (Fig.1). Specific IgG2b subclass antibody was not significantly increased against any of the epitopes.

Mice receiving rBCG018, did not produce any antibody responses against these epitopes. ESAT-6 and CFP-10 have previously been reported to be immunogenic in mice [24] and in non-human primates [25]. Mtb8.4 did not elicit significant IgG response in mice immunized with either rBCG018 or rBCG032, despite containing several B cell epitopes. Individual IgG subclass response against CFP-10 did not reach significant levels despite the elevated levels of total IgG produced against this epitope. We are uncertain why this discrepancy was observed but this may highlight the importance of measuring both total and subclass IgG to cater for subtle but significant changes in antibody response.

The subclass response observed against the individual B cell epitope suggest that both, the TH1 and TH2 arms of the immune response maybe operative in this study.

### Cellular immune response against T and B cell epitopes

#### Proliferative response against Ag85B T cell epitopes

Splenocytes isolated from rBCG018-immunized mice showed increased proliferative response after stimulation with Ag85B (P2) epitope (SI = 4.9); (P3) epitope (SI = 2.5) as well as with Mtb8.4 protein (SI = 3.2), whereas no response was observed against Ag85B (P1).

**Figure 2:**
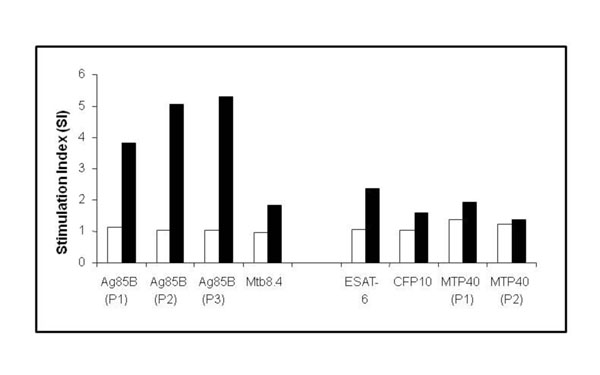
Lymphocyte proliferative response of rBCG032-immunized mice against the Ag85B T cell epitopes (P1, P2, P3); Mtb8.4; and the B cell epitopes of ESAT-6, CFP-10, and MTP40 (P1 and P2). The results are expressed as SI which is defined as the mean cpm of antigen-stimulated culture divided by the mean cpm of unstimulated culture. An SI > 2 is considered positive.

Splenocytes from rBCG032-immunized mice showed strong proliferative response to all the Ag85B T cell epitopes (P1, P2 and P3) with SI of 3.9, 5.0 and 5.3 respectively. There was no stimulation with Mtb8.4 protein. A moderate proliferative response was detected against the ESAT-6 B cell epitope (SI = 2.4) while all of the other B cell epitopes were not stimulatory (Fig. 2). Splenocytes stimulated with ConA proliferated vigorously (SI ≥ 20) (results not shown).

#### Intracellular cytokine expression against T and B cell epitopes and Mtb8.4 protein in animals immunized with rBCG018 or rBCG032

Table 1 summarizes the percentage of CD4+ and CD8+ lymphocytes expressing IL-2, IL-4 and IFN-γ and the “fold” difference between stimulated cells obtained from rBCG018 and rBCG032-immunized mice compared to those of BCG-immunized mice.

**Table 1:** Summary of the percentage of cells expressing selected cytokines as determined by flow cytometry. Response (fold increase) of CD4+ and CD8+ lymphocytes from rBCG-immunized mice (rBCG018, rBCG032) was compared to those of BCG-immunized mice (BCG).

CD4^+^/IL-2	Ag85B (P1)	Ag85B (P2)	Ag85B (P3)	Mtb8.4	ESAT-6	CFP10	MTP40 (P1)	MTP40 (P2)
BCG rBCG018 rBCG032	6.7 11.5 12.5	7.4 20.6* 16.5*	4.8 11.4* 6.5	3.8 12.5** 8.6*	6.1 3.6 5.6	3.1 2.4 8.4*	4.6 3.3 6.4	6.3 4.5 5.3

**CD4^+^/IL-4**								

BCG rBCG018 rBCG032	8.5 10.1 10.4	9.3 7.7 15.8	8.5 16.9 18.4*	9.4 8.4 13.8	3.5 1.1 3.8	5.4 3.5 5.7	8.4 7.8 8.6	5.9 5.7 8.4

**CD4^+^/IFN-γ**								

BCG rBCG018 rBCG032	4.6 17.4** 18.4**	8.0 21.5* 22.5*	6.4 16.6* 8.4	3.7 8.9* 7.5*	5.4 5.5 7.4	5.7 2.5 5.7	5.6 2.7 9.0	3.8 2.7 9.7*

**CD8^+^/IL-2**								

BCG rBCG018 rBCG032	12.3 21.5 25.0*	9.4 34.3** 30.5**	2.1 36.3** 30.4**	2.7 10.4** 9.8**	5.3 5.2 19.4**	3.6 6.4 18.3**	9.4 8.7 19.3*	4.7 5.5 7.4

**CD8^+^/IL-4**								

BCG rBCG018 rBCG032	5.5 16.5** 20.8**	3.7 15.5** 12.5**	7.8 16.5* 20.5*	5.7 8.4 13.8*	4.8 7.5 13.8*	4.6 8.6 11.4*	6.4 8.0 11.5	6.0 7.3 7.4

**CD8^+^/IFN-γ**								

BCG rBCG018 rBCG032	5.5 20.5** 25.4**	8.7 20.5* 24.5*	6.5 19.5** 21.5**	3.8 10.5* 9.7*	9.7 13.2 15.4	4.7 10.4* 11.3*	8.4 9.9 14.3	5.9 7.7 9.5

** ≥ 3 fold increase (high response) * ≥ 2 fold increase (moderate response) ≤ 2 fold increase (weak to no response)

We can conclude that some T cell epitopes may induce the production of Th2 instead of Th1 cytokines in Balb/c mice, and that the construction of a multi-epitope vaccine need to ensure that the overall potential “protection” that was intended is not compromised. In addition, it would be necessary to test the candidate vaccines in other strains of mice to ensure a more “universal” conclusion to be drawn.

The ability to elicit a predominant Th1 immune response is a desirable characteristic for any vaccine against MTB*.* The two vaccine candidates studied in the present work fulfilled this characteristic. In general, both rBCG018 and rBCG032 stimulated strong cellular response to the T cell epitopes and for the latter strong humoral immune response as well. Our findings suggest that co-expression of T and B cell epitopes of MTB in BCG stimulated both arms of the immune response although the protective capability of rBCG expressing both T and B cell epitopes needs further investigation.

## Competing interests

The authors declare that they have no competing financial interests.

## Authors' contributions

All authors have read and approved the final manuscript. RM and MA participated in recombinant strain production, immunogenicity studies, data analyses and in writing of the manuscript. DY and NA performed the bioinformatics studies. MES, AA and MNN conceived the study, the experimental design, bioinformatics studies, data analyses and in writing and finalizing of the manuscript.

## Declarations

This article has been published as part of *BMC Immunology* Volume 14 Supplement 1, 2013: Proceedings from Delivery Systems and Current strategies to drug design. The full contents of the supplement are available online at http://www.biomedcentral.com/bmcimmunol/supplements/14/S1.
